# Near-Infrared Spectroscopy Patterns as Indicator of Perioperative Stroke in Acute Type A Aortic Dissection

**DOI:** 10.3390/life15081295

**Published:** 2025-08-14

**Authors:** Henrik Heuer, André Truong, Christian Schach, Lukas Krämer, Jozef Micek, Franz Josef Putz, Bernhard Flörchinger, Fiona Rohlffs, Christof Schmid, Jing Li

**Affiliations:** 1 Department of Cardiothoracic Surgery, University Medical Center Regensburg, 93053 Regensburg, Germany; bernhard.floerchinger@ukr.de (B.F.); christof.schmid@ukr.de (C.S.); jing.li@ukr.de (J.L.); 2 Department of Vascular Surgery, University Medical Center Regensburg, 93053 Regensburg, Germany; andre.truong@ukr.de (A.T.); fiona.rohlffs@ukr.de (F.R.); 3 Department of Cardiology, University Medical Center Regensburg, 93053 Regensburg, Germany; christian.schach@ukr.de (C.S.); lukas.kraemer@ukr.de (L.K.); jozef.micek@ukr.de (J.M.); 4 Department of Nephrology, University Medical Center Regensburg, 93053 Regensburg, Germany; franz-josef.putz@ukr.de; 5 Department of Occupational Medicine, University Medical Center Regensburg, 93053 Regensburg, Germany

**Keywords:** type A aortic dissection, near infrared spectroscopy, cerebral oximetry, stroke, DHCA, malperfusion, neurologic outcome

## Abstract

Neurologic complications remain a major cause of morbidity in patients undergoing surgical repair of acute type A aortic dissection (ATAAD). Near-infrared spectroscopy (NIRS) is used for continuous, noninvasive monitoring of cerebral oxygenation during cardiopulmonary bypass; however, its utility in predicting perioperative stroke remains inadequately defined. A retrospective cohort study was conducted in 175 patients who underwent ATAAD repair between 2015 and 2023. Patients were stratified by the occurrence of perioperative stroke (*n* = 47, 26.9%). Intraoperative NIRS data, including cerebral regional oxygen saturation (crSO_2_) values at key procedural timepoints and signal variability with band power and crest factor, were analyzed in conjunction with demographic, anatomic, and postoperative variables. Patients with stroke exhibited significantly lower minimum NIRS values during deep hypothermic circulatory arrest (DHCA) (left: 46.7 (15.7–69.4) vs. 52.2 (22.0–81.6); right: 47.0 (23.3–78.5) vs. 56.3 (20.2–85.0); *p* = 0.03 and *p* < 0.01). Within the stroke group, NIRS signal variability was significantly greater (crest factor and standard deviation; *p* < 0.05) and showed blunted recovery post-DHCA. crSO_2_ values below 50% were more frequent in the stroke group (*p* = 0.04). Right common carotid artery dissection was more prevalent in the stroke group (40% vs. 23%, *p* = 0.04). ICU length of stay was significantly increased in patients with stroke. Cerebral desaturation and NIRS signal instability during DHCA are significantly associated with perioperative stroke in ATAAD repair. These findings support the prognostic value of intraoperative cerebral oximetry in detecting critical ischemic thresholds and identifying at-risk perfusion patterns.

## 1. Introduction

Acute type A aortic dissection (ATAAD) is a life threatening cardiovascular emergency requiring urgent surgical intervention to prevent death from rupture, tamponade, or malperfusion syndromes [1]. Despite advances in surgical techniques and perioperative care, stroke remains a devastating complication of ATAAD repair [2]. Reported rates of perioperative stroke range between 10% and 30%, contributing substantially to postoperative morbidity, prolonged hospitalization, and reduced quality of life in survivors.

Ensuring adequate cerebral protection during circulatory arrest remains a key challenge in aortic surgery [3]. Deep hypothermic circulatory arrest (DHCA), commonly used in complex arch procedures, provides neuroprotection by reducing metabolic demand but does not eliminate the risk of ischemic injury, particularly in the presence of cerebral malperfusion [4]. Adequate intraoperative monitoring of cerebral perfusion can be beneficial to adapt perfusion strategies and to detect neurological complications in evolution [5].

Near-infrared spectroscopy (NIRS) is a well-established, noninvasive modality to continuously monitor regional cerebral oxygen saturation (crSO_2_) during cardiac surgery [6]. By estimating the balance between cerebral oxygen supply and demand, NIRS provides a real-time surrogate for perfusion efficacy [7]. Its simplicity, safety, and bedside applicability have led to widespread application; however, its clinical utility—particularly in predicting neurologic outcomes in ATAAD—remains unclear. Although multiple studies have linked low intraoperative NIRS values to adverse outcomes in cardiac surgery broadly, fewer have specifically examined NIRS dynamics during DHCA in the context of ATAAD repair [8,9,10].

This study aimed to evaluate the relationship between intraoperative cerebral oximetry patterns and neurologic outcomes of surgical repair for ATAAD. Specifically, we sought to evaluate the following:

1. Determine whether intraoperative NIRS parameters—absolute minima, variability, and recovery—differ between patients with and without perioperative stroke.
2. Assess the association between preoperative vessel dissection status and cerebral oximetry patterns.
3. Gain insights into whether NIRS-derived variables may provide additive prognostic value to established risk scores such as the GERAADA score, which predicts 30-day mortality following ATAAD [11].

## 2. Materials and Methods

Study population: Between January 2015 and December 2023, 276 consecutive patients with ATAAD underwent open surgical treatment with intraoperative NIRS monitoring. A total of 25 patients were excluded due to preoperative neurological deficits, 19 patients due to preoperative mechanical ventilation, and 10 patients due to incomplete data.

NIRS Technology: The NIRS device (here INVOS 5100C, Medtronic, Northridge, LA, USA) uses light in the near-infrared spectrum (700–1000 nm) to penetrate biological tissues and measure the relative concentrations of oxygenated and deoxygenated hemoglobin within the cortical microvasculature. Optodes are bilaterally placed on the patient’s forehead, over the frontal cortex, to assess hemispheric cerebral oxygenation. The device applies spatially resolved spectroscopy using a light-emitting diode and two photodetectors at different distances from the source, enabling calculation of tissue oxygenation, predominantly in the venous compartment (~70% venous, ~25% arterial, ~5% capillary). Readings are updated every 5–6 s and displayed in real time [7].

**Figure 1:**
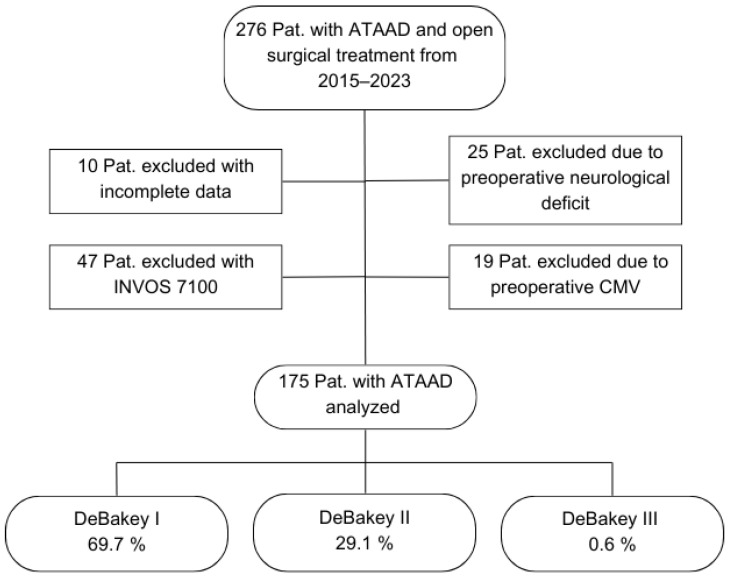
Flowchart of patient selection. Abbreviations: ATAAD, acute type A aortic dissection; CMV, controlled mechanical ventilation; Pat., patients.

In the course of using the newest INVOS version, INVOS 7100 system for ATAAD procedures from the year 2022 onwards, it became apparent that a potential pitfall of hemispheric misassignment evolved. The previous NIRS system 5100C defines the measured channel side only via the connection cable. The INVOS 7100 system, however, determines the measurement site via software, and the device also stores the individual ID of each sensor. If a laterality error is recognized, switching the connection cables does not correct the issue—only adjustment within the software can resolve it. In clinical practice, such mix-ups presumably occurred repeatedly. Although these were recognized and corrected intra-procedurally, we could not guarantee the accuracy of lateralization in the documentation. Due to this issue, this study includes only patients monitored using the INVOS 5100C system (Figure 1).

Study design and definitions: Patients with and without perioperative stroke were grouped and separately analyzed. Intraoperative NIRS values and rectal temperature as well as preoperative vessel dissection status were recorded in order to draw conclusions in regard to postoperative neurological outcomes. Perioperative stroke was defined as novel neurologic dysfunction with an imaging correlate in computer tomography (CT) or magnetic resonance imaging (MRI) during the course of the hospital stay. Vessel dissection was defined as a vessel which is affected by dissection in CT with or without clinical correlate.

Cardiopulmonary bypass (CPB) and deep hypothermic circulatory arrest (DHCA) with bilateral antegrade head perfusion were established with the main arterial cannula in the right subclavian artery. While no formal intervention algorithm or fixed desaturation thresholds were defined, the operating team continuously assessed NIRS values in real time and made individualized adjustments to optimize cerebral oxygenation. These adjustments included modifications in pump flow, perfusion pressure, temperature, and cannulation strategy, based on intraoperative trends. All decisions were made according to institutional best practices and clinical judgment.

Parameters and Features of NIRS: NIRS values were monitored via the oximeter INVOS 5100C (Medtronic, Dublin, Ireland). Values of the left and right (L/R) frontal hemisphere as well as the rectal temperature were recorded at five fixed measurement points (MP). MP1 was defined as 5 min before starting the CPB, MP2 as 1 min before DHCA, MP3 as the minimum value during DHCA, MP4 as 1 min post DHCA, and MP5 as 5 min before the end of CPB when the cardiac output is about the same as the CPB pump.

Several dynamic parameters were included in the analysis:

The band power was included, which equals a frequency spectrum, as the NIRS is seen as the signal: the higher and the more often changes in the sample values are observed, the higher the band power.

Mathematical Band Power = Sum of Squares of the Samples/Time.(1)

Here,

Band Power = (NIRS_MP2_^2^ + NIRS_MP3_^2^ + NIRS_MP4_^2^)/Duration_DHCA_. (2)

The crest factor depicts the fluctuations of the NIRS values. A larger spread between the extremes of the NIRS values corresponds to a higher crest factor.

Here,

(Max_NIRS_ − Min_NIRS_)/Mean_NIRS_. (3)

The NIRS Drop was defined as the relative difference between MP 1 and MP 3, and the NIRS Rise was defined as the relative difference between MP3 and MP5.

Statistical Analysis: The retrospective data were systematically extracted utilizing SAP (ERP 6.0, Germany) and subsequently compiled into a Libre Office spreadsheet (Libre Office 28.8.4.2, The Document Foundation, USA) in a pseudonymized format. Statistical analysis was performed with JupyterLab 4.2.5-1 (Project Jupiter, Emery County, UT, USA) running Python 3.9.6 (The Python Software Foundation, Beaverton, OR, USA). Continuous variables are presented as mean with a percentile-based 95% confidence interval. Binary parameters were reported as absolute and relative frequencies. Long-term survival analysis was conducted using the Kaplan–Meier method. *p*-values < 0.05 were considered statistically significant. A Student-t distribution was assumed due to unknown variance with a small sample size. The Mann–Whitney U-test was employed for continuous parameters with a significantly different median. Fisher’s Exact test was applied in dichotomous parameters. Bonferroni correction was skipped due to the small cohort size.

## 3. Results

### 3.1. Baseline Characteristics of the Study Population

The mean age of the included 175 patients was 60.8 (37.0–81.0) years. Of these patients, 68.0% were male, and 3.4% had a post-resuscitation status immediately prior to the surgical treatment. The mean preoperative GERAADA score indicating the predicted 30-day mortality in ATAAD patients was 21.5 (6.7–46.8). A total of 69.7% of the cases were classified as DeBakey Type I, 29.1% as DeBakey Type II, and 0.6% as DeBakey Type III. A total of 47 patients (26.9%) experienced a perioperative stroke, while 128 patients (73.1%) did not (Table 1).

### 3.2. Comparison of Stroke and No-Stroke

#### 3.2.1. Demographics and Preoperative Characteristics

There were no significant differences between the stroke and no-stroke groups in terms of age (60.5 (40.5–77.8) vs. 61.0 (37.0–81.8) years, *p* = 0.648) or sex distribution (32% female in both groups). However, the stroke group had a significantly higher GERAADA risk score (25.8 (10.7–49.9) vs. 19.9 (6.7–44.6), *p* = 0.003). Anatomical presentation differed slightly, with a higher prevalence of DeBakey I dissections among stroke patients (81% vs. 66%, *p* = 0.064) and significantly fewer DeBakey II dissections (17% vs. 34%, *p* = 0.039). Regarding the preoperative dissection status of the aortic branches—independent of clinical symptoms—dissection of the right common carotid artery (RCCA) and the right leg was more common in the stroke group (RCCA: 40% vs. 23%, *p* = 0.036; Right leg: 19% vs. 7%, *p* = 0.026) (Table 1).

#### 3.2.2. Intraoperative Data and Cerebral Oximetry

There were no significant differences in the duration of DHCA (47.7 (23.3–103.2) vs. 48.1 (18.0–108.6) min) or lowest rectal temperature (25.8 (21.3–29.0) vs. 25.5 (20.4–28.9) °C) between the groups. It is notable that the temperature management of all patients remained constant through the years and led to equivalent temperature values with low variance. However, notable differences were found in cerebral oximetry readings (Table 2).

During DHCA (MP3), patients who suffered a stroke had significantly lower minimum NIRS values (Left: 46.7 (15.7–69.4) vs. 52.2 (22.0–81.6), *p* = 0.027; Right: 47.0 (23.3–78.5) vs. 56.3 (20.2–85.0), *p* = 0.001). The crest factor of NIRS signals was significantly higher in the stroke group (Left: 0.52 (0.17–1.06) vs. 0.44 (0.11–1.01), *p* = 0.015; Right: 0.49 (0.17–1.01) vs. 0.41 (0.09–1.05), *p* = 0.016), suggesting greater signal variability. The standard deviation of all NIRS values was also elevated in stroke patients (12.7 (4.5–21.7) vs. 10.9 (3.6–22.8), *p* = 0.013).

A larger proportion of stroke patients had NIRS values fall below 50% at MP3 (68% vs. 49%, *p* = 0.039). Although the percentage drop from baseline (MP1 to MP3) did not reach conventional significance, trends were observed (e.g., Right hemisphere: 24 (−67–54) vs. 18 (−74–40), *p* = 0.060). Interestingly, the rebound in NIRS values post-DHCA to the end of CPB (MP3 to MP5) was significantly more pronounced in the no-stroke group in the right hemisphere (68 (−13–202) vs. 53 (−15–294), *p* = 0.011).

#### 3.2.3. Visualization of Near-Infrared Spectroscopy Dynamics

**Figure 2:**
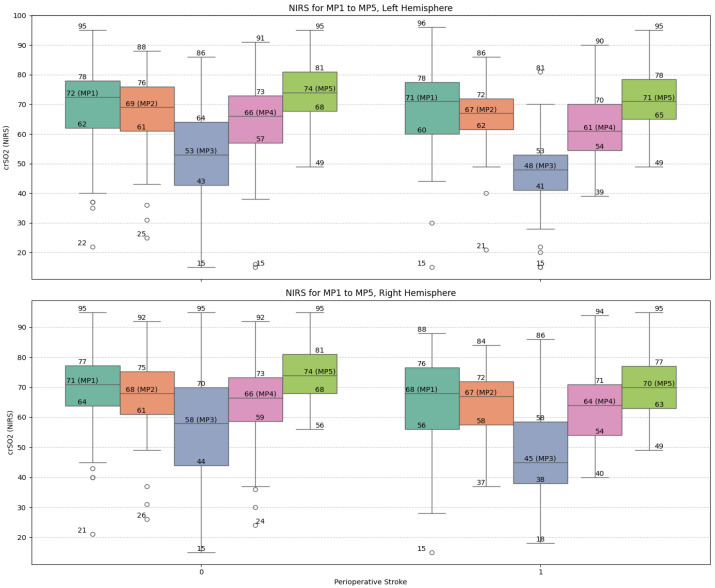
Box-plot visualization of NIRS values of the left and right frontal hemisphere and for the selected measure points MP 1 to MP 5: no-stroke: 0; stroke 1. Abbreviations: MP, measure point.

Figure 2 illustrates the trajectories of NIRS values across the five measurement points (MP1–MP5) for both hemispheres. The stroke group exhibited consistently lower values and greater variability, particularly around the nadir at DHCA (MP3), reinforcing the correlation between intraoperative cerebral desaturation and neurologic complications (Figure 2).

#### 3.2.4. Postoperative Outcomes

In 30% of patients with perioperative stroke, cerebral hypoxia contributed significantly to the cause of death during the course of the stay. Stroke patients had prolonged ICU stays (16.3 (3.1–48.2) vs. 10.0 (1.0–34.8) days, *p* = 0.001). However, total hospital stay and in-hospital mortality did not differ significantly between groups (Table 3).

#### 3.2.5. Survival Analysis

**Figure 3:**
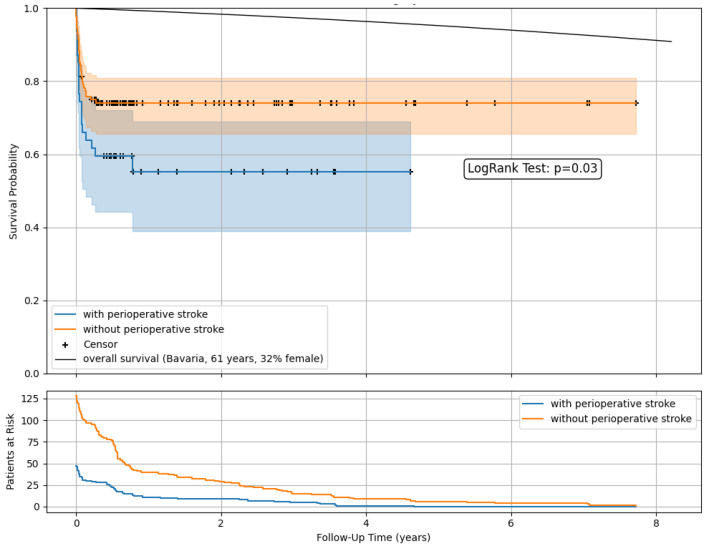
Postoperative survival and follow-up data after open surgery in ATAAD with and without perioperative stroke. Kaplan–Meier survival estimates with censoring. Comparison with the overall population survival as imposed by official data.

Kaplan–Meier survival estimates (Figure 3) demonstrated lower postoperative survival rates among patients with perioperative stroke compared to those without. A comparison with the overall population survival as imposed by official data was included [12].

At a mean follow-up of 493 days, 47 patients with and without stroke (27%) were in observation. The 1-year survival was 55.3% vs. 74.1%, with 11 vs. 40 patients at risk and remaining stable in the following years.

## 4. Discussion

### 4.1. State of Research

Seminal work by Orihashi et al. (2004) first suggested that NIRS could detect cerebral malperfusion and serve as an early indicator of neurologic injury [8]. In comparison to conventional modalities such as electroencephalography, which requires expert interpretation, or transcranial Doppler, which is operator-dependent and intermittent, NIRS—as a continuous, noninvasive, real-time monitoring of cerebral oxygenation—holds advantages, thus facilitating timely recognition of cerebral hypoperfusion, especially during DHCA [8,9,13]. More recently, Gaudino et al. (2020) and Bochmann et al. (2022) highlighted the prognostic utility of intraoperative cerebral oximetry across aortic procedures [9,13]. Notably, these studies emphasized that not only absolute desaturation thresholds but also signal variability may reflect cerebral hemodynamic instability and portend neurologic insult. Complementing these findings, emerging studies such as those by Hibino et al. (2024), Pierik et al. (2024), and Kletzer et al. (2024) are now exploring the integration of NIRS with clinical risk models and perfusion strategies to refine perioperative risk assessment [10,14,15].

Despite these advances, significant gaps remain. It is unclear which NIRS parameters—minimum value, rate of decline, signal variability, or recovery slope—are most predictive of stroke. Furthermore, the relationship between NIRS dynamics and preoperative factors, such as vessel dissection status and anatomical subtype (e.g., DeBakey classification), is not fully elucidated. Additionally, there is limited evidence on whether NIRS-guided interventions can reduce the incidence or severity of neurologic complications in the setting of ATAAD surgery.

### 4.2. Summary of Main Findings

In this cohort of 175 patients undergoing surgical repair for acute type A aortic dissection (ATAAD), perioperative stroke occurred in 26.9% of cases. Patients who suffered stroke exhibited the following:

- Higher preoperative GERAADA scores;
- More frequent dissection of the RCCA and the right lower limb;
- Significantly lower cerebral oximetry minima during DHCA and a muted post-DHCA recovery;
- Greater variability in NIRS signal (higher crest factor and standard deviation);
- Increased incidence of cerebral hypoxia as cause of death in the postoperative course and prolonged ICU stays.

These findings strongly support the hypothesis that intraoperative cerebral desaturation and impaired NIRS dynamics during DHCA are associated with neurologic complications and adverse clinical outcomes in ATAAD repair.

### 4.3. Cerebral Desaturation and Neurologic Injury

The extensive literature has established a link between intraoperative cerebral desaturation and neurologic injury in cardiac surgery. Gaudino et al. (2020) conducted a meta-analysis demonstrating that declines in intraoperative cerebral oximetry correlate with postoperative neurologic deficits across various cardiac procedures [13]. Our data align with these findings: stroke patients experienced markedly lower NIRS minima during DHCA (MP3). These results reinforce that cerebral hypoperfusion during circulatory arrest remains a critical risk factor—especially under deep hypothermia, which is intended to reduce ischemic insult. The muted NIRS rebound in patients with stroke suggests sustained microvascular or metabolic impairment after reperfusion, consistent with Bochmann et al. (2022), who reported that inadequate recovery of cerebral oxygenation post-CPB predicted worse neurologic outcomes in complex aortic surgery [9]. The right hemisphere’s diminished reoxygenation highlights possibly asymmetrical perfusion recovery, potentially related to variations in bypass cannulation or residual malperfusion—a hypothesis warranting further anatomical and functional mapping in future studies. One important consideration is that although continuous NIRS monitoring influenced intraoperative decision-making, no standardized thresholds or protocols were employed to guide interventions. Adjustments were made dynamically and based on the clinical judgment of the surgical and perfusion team. This individualized approach reflects current clinical reality but limits the reproducibility and standardization of results. Future prospective studies may benefit from protocolized responses to defined NIRS thresholds to clarify the causal impact of targeted interventions.

### 4.4. Signal Variability: Beyond Absolute Oximetry Values

Elevated crest factor and standard deviation in patients with stroke point to increased cerebral oximetry variability, potentially reflecting unstable cerebral perfusion or fluctuating metabolic demands during DHCA and reperfusion. Orihashi et al. (2004) suggested that cerebral blood flow instability, detected by NIRS, precedes neurologic injury [8]. Our findings reinforce this concept: not only lower absolute minima are significant, but greater variability—indicative of transient ischemic episodes due to fluctuations in perfusion pressures or autoregulatory failure—may increase vulnerability to neurologic injury [16,17]. These dynamic metrics could complement absolute thresholds to identify high-risk intraoperative patterns.

### 4.5. Malperfusion Syndromes and Stroke Risk

Dissection involving the RCCA was significantly more prevalent in patients with stroke (40.4% vs. 23.4%, *p* = 0.04). Similarly, increased dissection involving the right leg (19.1% vs. 7.0%, *p* = 0.03)—indicative of widespread aortic branch involvement—was noted. These findings align with Pierik et al. (2024), who reported that preoperative carotid dissection independently increased stroke risk in ATAAD [10]. Studies by Vendramin et al. (2022) and Kreibich et al. (2019) similarly highlighted the prognostic impact of carotid and cerebral malperfusion [18,19]. Malperfusion compromises baseline cerebral oxygen delivery, exacerbating the effects of intraoperative hypoperfusion. Routine assessment of carotid flow (via duplex or CT angiography) and electroencephalographic monitoring could supplement NIRS to stratify risk and guide cannulation strategies.

### 4.6. GERAADA Risk Score Validation

Patients with stroke had higher GERAADA scores (25.8% vs. 19.9%), indicating more severe preoperative risk. Although the GERAADA score was developed to predict mortality and operative risk, its inclusion of preoperative shock, tamponade, and malperfusion syndromes captures factors predisposing to cerebral injury [20]. Kletzer et al. (2024) recently emphasized GERAADA’s correlation with stroke incidence post-ATAAD surgery [14]. In multivariate modeling that includes intraoperative NIRS, GERAADA could improve prognostic accuracy—an avenue that merits formal testing.

### 4.7. Postoperative Course and Intensive Care Unit Utilization

In 30% of patients with stroke, cerebral hypoxia contributed significantly to the recorded mortality, and patients with stroke stayed significantly longer in the ICU (16.3 vs. 10 days). This trend parallels Hibino et al. (2024), who found that cerebral oximetry abnormalities were associated not only with stroke but also with delayed recovery, prolonged mechanical ventilation, and increased ICU length of stay [15]. The data reinforce that stroke drives a cascade of complications, promoting resource utilization and poorer outcomes. Early detection of NIRS deterioration and swift corrective action may lessen ICU burden, but prospective interventional trials are needed to confirm this.

### 4.8. Implications for Cerebral Protection Strategies

Our findings have direct implications for cerebral protection during ATAAD repair:

- More proactive NIRS thresholds: Our data suggest that thresholds below ~50% during DHCA are highly predictive of stroke. Early deepening of hypothermia and augmentation of cerebral perfusion pressure may prevent critical declines with hypoxia [21].
- Real-time signal variability monitoring: Continual tracking of dynamic parameters, e.g., crest factor and standard deviation, may alert surgeons to impending perfusion instability. Automated alert algorithms could be developed to prompt intervention before desaturation worsens. NIRS-guided perfusion adjustments have already demonstrated efficacy in other high-risk cardiac surgeries [22].

These strategies echo recommendations by Bochmann et al. (2022), who endorse dynamic NIRS-guided perfusion adjustments during aortic arch surgery [9].

### 4.9. Biological Plausibility and Mechanisms

Several physiological mechanisms may explain our observations:

1. Pre-existing Hypoperfusion: Patients with carotid malperfusion have diminished cerebral reserve, rendering any intraoperative hypoxia more harmful.
2. DHCA-Induced Ischemia: Even with deep hypothermia, metabolic suppression fails to entirely prevent neural injury, particularly in marginally perfused regions.
3. Reperfusion Injury: Cerebral desaturation rebound is limited in stroke patients, suggesting endothelial dysfunction or microthrombosis that compromise reperfusion.
4. Microembolism: Fluctuating NIRS might reflect embolic showers during circulatory arrest and restoration, e.g., through aortic manipulation [23].

Orihashi et al.’s study (2004) and subsequent work have documented NIRS dips attributable to both hypoperfusion and embolism, reinforcing the multifactorial nature of injury [8,13].

### 4.10. Integration of Autoregulation Monitoring and Dynamic NIRS Assessment

These findings also align with a growing interest in the use of NIRS not only for static saturation thresholds but also for mapping cerebral autoregulation in real time. Sainbhi (2023) introduced a noninvasive protocol to dynamically assess cerebral autoregulation using near-infrared spectroscopy, aiming to delineate patient-specific optimal perfusion pressures during cardiac surgery [24]. This approach shifts the role of NIRS from passive monitoring toward active, physiology-driven guidance of hemodynamics. In our cohort, the increased NIRS signal variability and elevated crest factor in patients with stroke may reflect episodes of impaired autoregulation—intervals during which the brain is unable to buffer changes in perfusion pressure, increasing susceptibility to ischemic injury. Integrating continuous autoregulation indices, as proposed by Sainbhi, could improve the interpretation of intraoperative NIRS patterns by differentiating tolerable fluctuations from pathologic instability. Moreover, integrating autoregulatory thresholds could enable the surgical team to titrate flow or pressure in real time, potentially preventing prolonged desaturation episodes. Future studies should investigate whether combining absolute crSO_2_ targets with autoregulation-guided perfusion strategies can reduce the incidence of neurologic complications in ATAAD repair.

### 4.11. Study Limitations

While robust, our study has limitations: First, it is retrospective and single-center in design, limiting generalizability despite a standardized surgical protocol. Second, the perioperative stroke definition may underrepresent minor or subclinical neurocognitive deficits; serial neuropsychological testing would enhance outcome granularity. Third, while NIRS offers continuous and noninvasive monitoring of cerebral oxygenation, it is limited to frontal cortical regions. Adding transcranial Doppler or cerebral oximetry of other regions could improve detection of deep brain ischemia.

Importantly, although the perfusion strategy was standardized with BACP during DHCA in all patients, this uniformity precludes comparison with alternative techniques. However, it strengthens internal consistency and isolates NIRS metrics as more independent predictors of neurologic outcomes. Although this was an observational study, continuous NIRS monitoring did inform intraoperative decision-making. However, no predefined NIRS thresholds were used to trigger specific interventions, and adjustments were made at the discretion of the surgical team. This limits reproducibility and causal inference. Multivariable modeling with logistic regression was tried; however, due to sample size constraints, in particular limited stroke events and insufficient variance also across covariates (including GERAADA score and RCCA dissection), no stable or interpretable model could be generated. Lastly long-term neurologic outcomes were not assessed.

Future prospective, multicenter studies with structured neurologic assessment, dynamic perfusion mapping, and interventional NIRS protocols are warranted to validate these findings.

## 5. Conclusions

Intraoperative cerebral desaturation and increased variability in NIRS signals during DHCA are significantly associated with perioperative stroke in patients undergoing surgical repair for acute type A aortic dissection. These findings highlight the prognostic value of cerebral oximetry—particularly dynamic parameters—in identifying patients at risk for neurologic injury. Incorporating real-time NIRS monitoring into standardized perfusion protocols may enhance cerebral protection strategies and improve outcomes in aortic surgery.

## Figures and Tables

**Table 1:** Demographic data and preoperative status.

Cohort 2015–2023	Total	Stroke	No-Stroke	*p*-Value	Rating
Patients	175 (100)	47 (100)	128 (100)	-	-
Age at procedure	60.8 (37.0–81.0)	60.5 (40.5–77.8)	61.0 (37.0–81.8)	0.647	-
Female	56 (32.0)	15 (31.9)	41 (32.0)	1.000	-
DeBakey I	122 (69.7)	38 (80.9)	84 (65.6)	0.064	*
DeBakey II	51 (29.1)	8 (17)	43 (34)	0.039	**
DeBakey III	1 (0.6)	0 (0.0)	1 (0.8)	1.000	-
DeBakey other	1 (0.6)	1 (2.1)	0 (0.0)	0.269	-
GERAADA-Score (%)	21.5 (6.7–46.8)	25.8 (10.7–49.9)	19.9 (6.7–44.6)	0.002	***
Total Arch Replacement	20 (11.4)	5 (10.6)	15 (11.7)	1.000	-
Vessel dissection:					
coronary	11 (6.3)	5 (10.6)	6 (4.7)	0.168	-
innominate	58 (33.1)	21 (44.7)	37 (28.9)	0.069	*
RCCA	49 (28.0)	19 (40.4)	30 (23.4)	0.036	**
LCCA	42 (24.0)	10 (21.3)	32 (25.0)	0.693	-
RSA	31 (17.7)	10 (21.3)	21 (16.4)	0.504	-
LSA	32 (18.3)	8 (17.0)	24 (18.8)	1.000	-
spinal	4 (2.3)	2 (4.3)	2 (1.6)	0.292	-
coeliacal	48 (27.4)	16 (34.0)	32 (25.0)	0.255	-
SMA	29 (16.6)	9 (19.1)	20 (15.6)	0.647	-
RRA	28 (16.0)	11 (23.4)	17 (13.3)	0.110	-
LRA	73 (41.7)	21 (44.7)	52 (40.6)	0.730	-
Leg R	18 (10.3)	9 (19.1)	9 (7.0)	0.026	**
Leg L	29 (16.6)	11 (23.4)	18 (14.1)	0.169	-
CPR, prae procedure	6 (3.4)	3 (6.4)	3 (2.3)	0.345	-

Data presented as [*n* (%)] or [mean (CI 95%)]. *** *p* < 0.01/** *p* < 0.05/* *p* < 0.1. Abbreviations: CPR, cardiopulmonary resuscitation; LCCA, left common carotid artery; LRA, left renal artery; RCCA, right common carotid artery; RRA, right renal artery; SMA, superior mesenteric artery.

**Table 2:** Intraoperative data and NIRS analysis.

	Total	Stroke	No-Stroke	*p*-Value	Rating
Duration of DHCA (minutes)	48.0 (18.3–106.3)	47.7 (23.3–103.2)	48.1 (18.0–108.6)	0.815	-
Lowest Rectal Temperature (°C)	25.6 (20.7–29.0)	25.8 (21.3–29.0)	25.5 (20.4–28.9)	0.585	-
Temperature (°C) at					
MP1—rectal, pre-CPB	36.2 (34.1–37.7)	36.1 (33.9–37.7)	36.2 (34.2–37.7)	0.398	-
MP2—rectal, pre-DHCA	27.1 (21.9–33.0)	27.1 (23.1–32.1)	27.1 (21.9–33.7)	0.948	-
MP3—head temp at DHCA	21.2 (18.0–28.0)	21.6 (20.0–28.0)	21.0 (18.0–28.0)	0.075	*
MP4—rectal, post-DHCA	26.6 (22.2–31.0)	26.6 (23.3–30.9)	26.5 (22.2–30.9)	0.957	-
MP5—rectal, end of CPB	35.5 (31.7–37.3)	35.4 (30.9–37.2)	35.6 (32.5–37.5)	0.808	-
NIRS (Left/Right) at					
MP1—pre-CPB	69.3 (37.0–92.6) 68.9 (40.0–89.6)	67.6 (32.1–82.0) 65.6 (29.5–82.7)	69.9 (37.5–93.3) 70.1 (43.3–91.6)	0.459	-
				0.114	-
MP2—pre-DHCA	67.2 (41.0–86.0) 67.6 (49.3–89.6)	65.9 (41.3–79.8) 65.5 (50.3–82.8)	67.7 (43.5–86.0) 68.4 (49.2–90.0)	0.337	-
				0.117	-
MP3—minimum at DHCA	50.7 (16.7–80.6) 53.8 (20.3–85.0)	46.7 (15.7–69.4) 47.0 (23.3–78.5)	52.2 (22.0–81.6) 56.3 (20.2–85.0)	0.027	**
				0.001	***
MP4—post-DHCA	63.6 (40.3–85.6) 65.4 (39.3–89.0)	61.7 (40.3–84.8) 63.5 (42.7–87.0)	64.3 (41.2–84.6) 66.1 (37.3–89.0)	0.100	-
				0.109	-
MP5—end of CPB	73.2 (54.4–90.3) 73.6 (55.3–93.0)	71.7 (53.0–93.9) 70.8 (50.4–93.4)	73.7 (57.2–88.8) 74.6 (58.2–92.6)	0.159	-
				0.044	**
Crest Factor of NIRS values (L/R)	0.46 (0.12–1.01) 0.43 (0.10–1.05)	0.52 (0.17–1.06) 0.49 (0.17–1.01)	0.44 (0.11–1.01) 0.41 (0.09–1.05)	0.015	**
				0.016	**
Band Power of NIRS values (L/R) at DHCA (1/min)	289 (77–675) 303 (89–675)	262 (75–508) 272 (76–604)	298 (89–714) 315 (120–680)	0.400	-
				0.127	-
SD of all NIRS samples	11.4 (3.9–22.6)	12.7 (4.5–21.7)	10.9 (3.6–22.8)	0.013	**
NIRS MP3 < 50%	95 (54.3)	32 (68.1)	63 (49.2)	0.039	**
NIRS Drop MP1-MP3 (%, L/R)	−23 (−74–40) −19 (−72–41)	−23 (−74–40) −24 (−67–54)	−23 (−73–25) −18 (−74–40)	0.089	*
				0.060	*
NIRS Drop MP1-MP3 > 20%	116 (66.3)	35 (74.5)	81 (63.3)	0.207	-
NIRS Rise MP3-MP5 (%, Left/Right)	61 (−01–277) 0.57 (−15–289)	72 (9–283) 68 (−13–202)	57 (−1–244) 53 (−15–294)	0.115	-
				0.011	**
NIRS Rise MP3-MP5 > 20%	138 (78.9)	41 (87.2)	97 (75.8)	0.143	-
NIRS Drop and Rise > 20%	108 (61.7)	34 (72.3)	74 (57.8)	0.114	-

Data presented as [mean (CI 95%)] or [*n* (%)]. *** *p* < 0.01/** *p* < 0.05/* *p* < 0.1. Abbreviations: DHCA, deep hypothermic circulatory arrest; MP, measure point; NIRS, noninvasive infrared spectroscopy.

**Table 3:** Postoperative course.

	Total	Stroke	No-Stroke	*p*-Value	Rating
EL due to Cerebral Hypoxia	15 (8.6)	14 (29.8)	1 (0.8)	0.000	***
Days on ICU	11.7 (1.0–46.6)	16.3 (3.1–48.2)	10.0 (1.0–34.8)	0.000	***
Days in Clinic	21.1 (2.3–57.6)	23.5 (4.0–59.8)	20.2 (2.0–57.0)	0.254	-
In-Hospital Mortality	32 (18.3)	12 (25.5)	20 (15.6)	0.184	-

Data presented as [mean (CI 95%)] or [*n* (%)]. *** *p* < 0.01. Abbreviations: EL, exitus letalis, ICU, intensive care unit.

## Data Availability

The data presented in this study are available from the corresponding author upon reasonable request.

## Abbreviations

The following abbreviations are used in this manuscript:

ATAAD	Acute type A aortic dissection
BACP	Bilateral antegrade cerebral perfusion
CBP	Cardiopulmonary bypass
CT	Computer tomography
DHCA	Deep hypothermic circulatory arrest
EL	Exitus letalis
GERAADA	German registry for acute aortic dissection type A
ICU	Intensive care unit
LCCA	Left common carotid artery
LRA	Left renal artery
MAP	Mean arterial pressure
MRI	Magnetic resonance imaging
NIRS	Near infrared spectroscopy
RCCA	Right common carotid artery
RRA	Right renal artery
SACP	Selective antegrade cerebral perfusion
SD	Standard deviation
SMA	Superior mesenteric artery
S/P	Status post
